# Novel abrasive-free jet polishing for Bulk single-crystal KDP with a low viscosity microemulsion

**DOI:** 10.1038/s41598-022-12447-3

**Published:** 2022-05-18

**Authors:** Yan Zhang, Qichao Fan, Wei Gao, Chao Wang, Fang Ji

**Affiliations:** Institute of Machinery Manufacturing Technology, China Academy of Engineering Physics, Mianyang, 621900 China

**Keywords:** Chemical engineering, Mechanical engineering

## Abstract

In present work, the abrasive-free jet polishing (AFJP) of bulk single-crystal KDP was first fulfilled, when using a newly-designed low-viscosity microemulsion as the AFJP fluid. The novel AFJP fluid shows a typical water-in-oil structure, in which the water cores uniformly distribute in the BmimPF6 IL, with a particle size of about 20–25 nm. What’s more, the AFJP fluid is a controllable and selective non-abrasive jet fluid that the shape of the removal function is regular and smooth, presenting a similar Gaussian function, meanwhile, the dispersion coefficient of the removal rate is only 1.9%. Finally, the surface quality of the bulk single-crystal KDP is further improved by AFJP, meanwhile, the subsurface damage is first obviously mitigated.

## Introduction

Bulk single-crystal potassium dihydrogen phosphate (KDP) is a unique single crystal material with excellent optical properties, such as high nonlinear conversion efficiency, superior photoelectric and piezoelectric properties^1–3^, and thus has been chosen as the key optical material of inertial confinement fusion (ICF) facility which is regarded as the future of nuclear energy^4–6^. However, KDP is very difficult to cut and polish, because the optical material is crisp, soft and easy to dissolute at atmosphere^7–9^. Currently, the only practical precise KDP machining technique is single-point diamond turning (SPDT)^10–12^. However, this method inevitably generates some microcosmic grooves, scratches and cracks on the crystal surface, and thus deteriorates the optical properties of KDP^13–15^.

In order to improve the surface quality, many polishing methods have been developed for KDP ultra-precision, such as magnetorheological finishing (MRF)^16–18^, ion-beam figuring (IBF)^19,20^, and chemical mechanical polishing (CMP)^21,22^. As to MRF, the magnetic particles are easy to embed into the crisp and soft KDP surface and thus cause secondary pollution^18^. As compared to MRF, the IBF and CMP are particle-free polishing methods. However, IBF usually generates a temperature gradient field on the KDP surface, and thus causes thermal cracks^19,20^, while CMP is hard to ensure a good surface uniformity for large-size KDP because of the high viscosity of the polishing fluid^21,22^.

Recently, we have developed an abrasive-free jet polishing (AFJP) method for the mitigation of subsurface damage caused by SPDT, using a water-in-oil (w/o) type microemulsion as the AFJP fluid, which contains large amounts of nanoscale water-cores evenly dispersed in the non-aqueous carrier ionic liquid (IL)^23–25^. The addition of a long-chain surfactant is essential for forming nanoscale water-cores in IL, since it decreases the surface energy between water and IL phases^26^. In the static state, the water-cores are separated from the KDP by the barrier of the long-chain surfactant and IL. During the AFJP process, the barrier is broken by the impact press, and then the water-cores in IL can contact and remove KDP through dissolution at the interface. Generally speaking, the novel AFJP provides a new way addressing the ultra-precision KDP polishing, meeting the requirements of high-energy laser systems.

In previous study, the common 1-butyl-3-methylimidazolium hexafluorophosphate (Bmim[PF6]) and Triton X-100 (TX-100) have been chosen as the IL and surfactant of the microemulsion H_2_O/TX-100/Bmim[PF6] (BF), respectively^23–25^. The common microemulsion BF first demonstrates the novel AFJP method of polishing KDP crystal. However, due to its extremely high viscosity, 215mpa·s at room temperature (RT), the BF is easily mixed with air which produces large amounts of bubbles, and thus significantly affects the stability of removal function, limiting the practical application of AFJP in polishing large-size KDP crystal.

In present work, a new type of low-viscosity microemulsion is designed as the AFJP fluid, under the premise of good compatibility with KDP. Because of its low viscosity, the AFJP fluid effectively addresses the problem of bubble generation during the polishing process, which obviously improves the stability of the removal function, thereby first fulfills the practical application of AFJP in large-size KDP ultra-precise polishing.

## Methods

**Figure 1:**
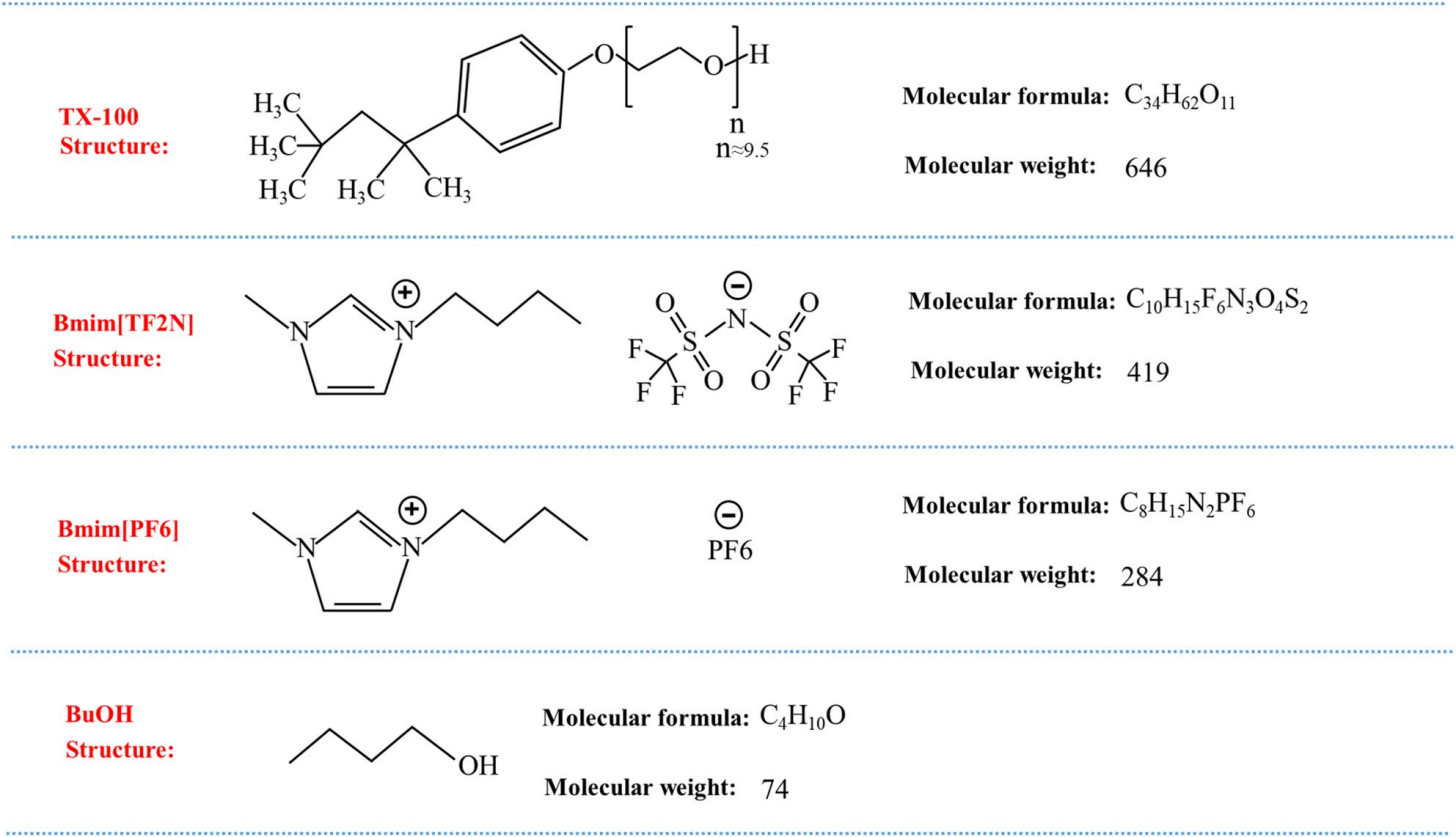
The molecular structure, formula and weight of TX-100, Bmim[TF2N], Bmim[PF6] and BuOH.

The microemulsions were synthesized at a constant environment temperature 25 °C. The original materials, Bmim[TF2N], Bmim[PF6], TX-100 surfactant and BuOH co-surfactant, with a purity higher than 99%, were all bought from Sigma Aldrich. The molecular structure, formula and weight of these original materials are shown in Fig. 1. The water is deionized water. As shown in Table 1, four kinds of microemulsions were prepared by mixing above pure materials, and expressed as follows: H_2_O/TX-100/Bmim[PF6] (BF), H_2_O/TX-100/Bmim[TF2N] (BT1 BT2 and BT3), H_2_O/TX-100:BuOH/Bmim[TF2N] (BT + BuOH) and H_2_O/TX-100:BuOH/Bmim[PF6] (BF + BuOH). The viscosity of these microemulsions was measured by a rotational viscometer (Brookfield Model: LVDV-II + P). During the viscosity measurement, a calibrated beryllium-copper spring was used to drive a rotor to continuously rotate in the fluid, while the torque was measured by a rotational torque sensor. The torque is proportional to the viscosity of the liquid. The BF, BT1, BT2 and BT3 were tested using SC4-34 (sample volume 10 mL, measurement range 30 ~ 600 K mpa.s) rotors, while the BF/BT1 + BuOH were tested using SC4-18 (sample volume 8 mL, measurement range 1.5 ~ 30 K mpa.s) rotors. The initial speed was set to 100 rpm, while the speed increment and cycle number were set to 5 rpm/min and 20, respectively. The testing temperature was set to 25 °C. The ambient humidity was 35%. The data were collected by an external display and recording device, with a collecting interval of 30 s.

**Table 1:** Microemulsion constitutions.

Name	Microemulsion constitution	Water content (wt%)	From
BF	H_2_O/TX-100/Bmim[PF6]	2	^23–25^
BF + BuOH	H_2_O/TX-100:BuOH/Bmim[PF6]	2	This work
BT1	H_2_O/TX-100/Bmim[TF2N]	2	This work
BT2	H_2_O/TX-100/Bmim[TF2N]	3	This work
BT3	H_2_O/TX-100/Bmim[TF2N]]	5	This work
BT1 + BuOH	H_2_O/TX-100:BuOH/Bmim[TF2N]	2	This work

Meanwhile, the compatibility between these microemulsions and KDP was conducted by placing the microemulsions on the KDP surface for 14 h, and then the KDP surface was observed using an optical microscope. The cryo-transmission electron microscope (cryo-TEM FEI Talos Arctica) was used to visually confirm the microemulsion structure. A self-designed AFJP device was used to conduct the multi-point experiment and polishing experiment of bulk single-crystal KDP^25^. The multi-point experiment was performed to evaluate the removal controllability and stability of the newly-designed low-viscosity microemulsion (AFJP fluid) BT1, BT2 and BT3, with the praying pressure, distance and nozzle of 0.5 MPa, 10 mm and 1 mm, respectively. The morphology feature was detected using a laser interferometer (ZIGO, λ = 632 nm). The spraying time of BT1, BT2 and BT3 is 5 min, 1 min and 10 s, respectively, making sure of that the spot depth is between the testing range of the laser interferometer. The AFJP polishing experiment was conducted on the (010) surface of the bulk single-crystal KDP using the newly-designed low-viscosity AFJP fluid BT1. Before polishing experiments, the (010) surface was precisely machined using SPDT. The spindle speed, cutting depth and feed rate of SPDT were 280 r/min, 4 μm and 4 mm/min, respectively. After machining, the (010) SPDT surface was polished by AFJP using the newly-designed low-viscosity AFJP fluid BT1. The scanning area, scanning speed, step distance and number of scans were 35 mm × 35 mm, 0.05 mm/s, 0.5 mm and 4, respectively. The spraying pressure, distance and nozzle were 0.5 MPa, 10 mm and 1 mm, respectively. The morphology feature before and after AFJP was detected using the laser interferometer (ZIGO, λ = 632 nm). The structure of subsurface damage before and after AFJP was evaluated using the X-ray grazing incidence technique (GIXRD) by a Bruker D8 Discover XRD apparatus with Cu Ka radiation. The scan range (2θ), step size and counting time of GIXRD were 15° ~ 90°, 0.05° and 1.32 s, respectively.

## Results and discussion

### Designing of low-viscosity AFJP fluid

**Figure 2:**
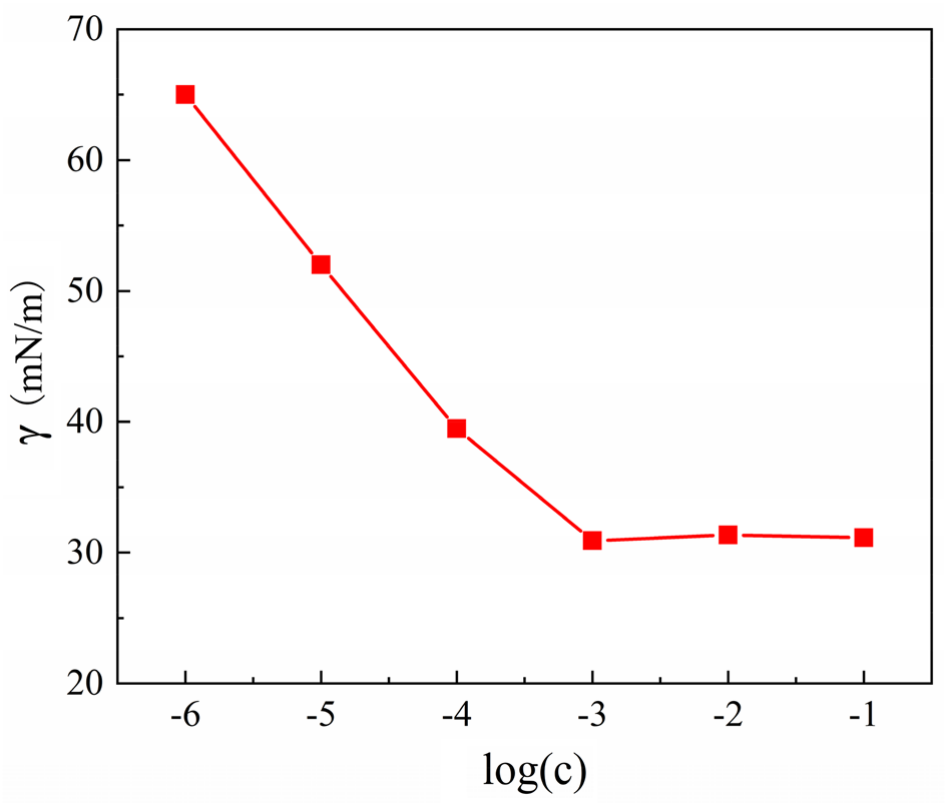
The relationship between the surface tension (γ mN/m) and concentration (c mol/L).

Microemulsion is an optically isotropic, thermodynamically and kinetically stable liquid solution, consisting of oil, water and a surfactant. In our previous work, the common microemulsion BF first demonstrates the novel AFJP method of polishing KDP crystal^23–25^. The dispersion type of the microemulsion BF is water-in-oil, where Bmim[PF6] IL acts as the oil to carry the nanoscale water-cores, while TX-100 acts as the surfactant to decrease the surface energy between BmimPF6 IL and water, thus promoting the formation of nanoscale water-cores^23–25^. Figure 2 presents the surface tension of TX-100 with different concentrations. Shown in the figure, when the surfactant concentration is low, the surface tension decreases sharply with increasing the concentration. The surface tension reaches a steady state with further increasing the concentration after reaching the critical micelle concentration (CMC), which is the minimum concentration required to form micelles^27–29^. The CMC is determined to be about 0.001 mol/L.

**Figure 3:**
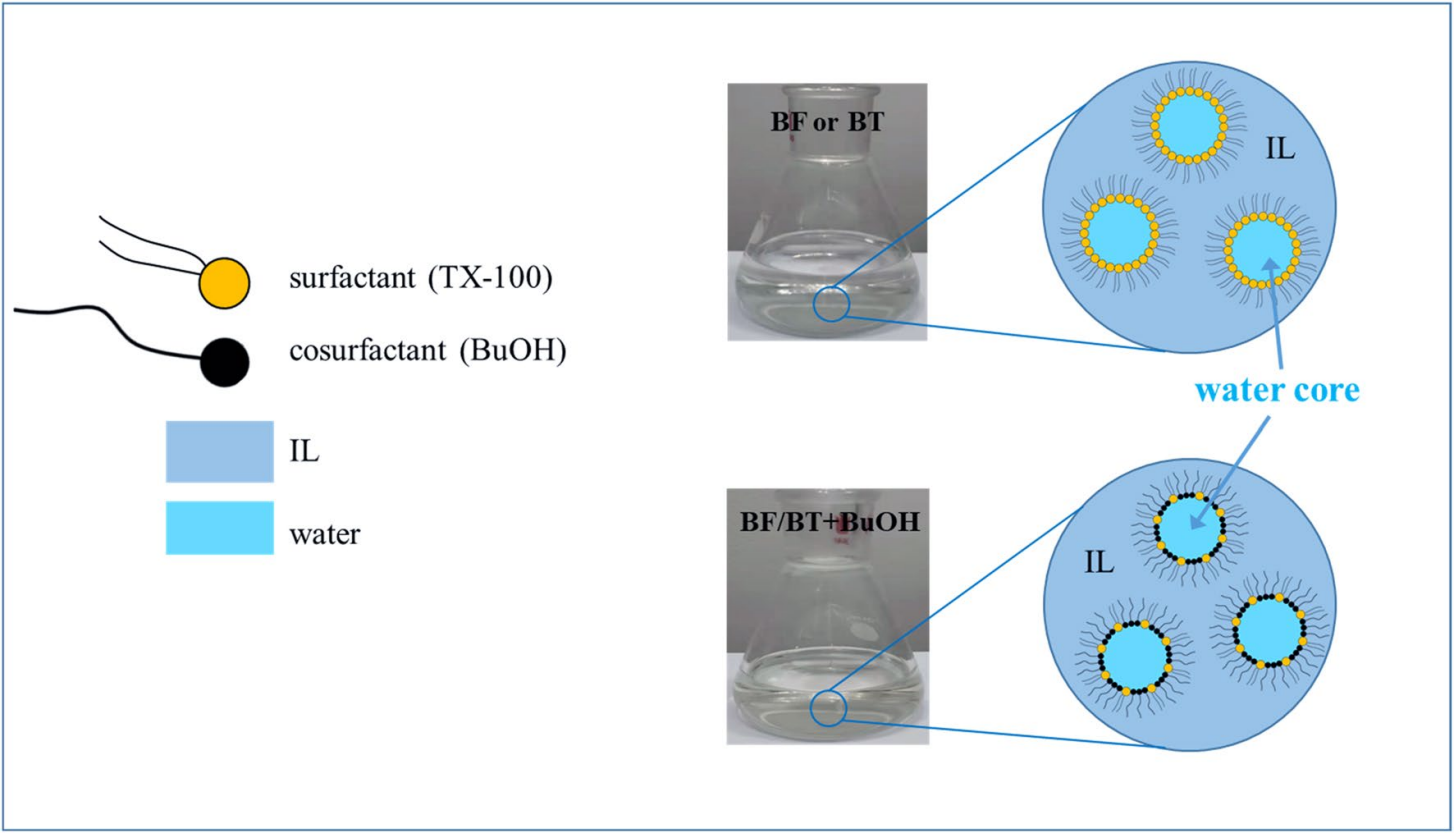
Schematic illustration of the w/o IL microemulsion structure.

**Figure 4:**
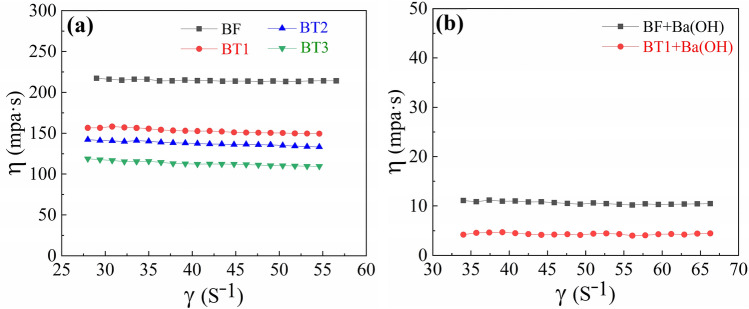
The viscosity of the microemulsions (BF, BT1, BT2, BT3, BF + Ba(OH) and BT1 + Ba(OH)).

However, the microemulsion BF presents an extremely high viscosity of 215mpa·s at room temperature, and thus limiting the practical application of AFJP in polishing large-size KDP crystal. Previously, Wasserscheid et al. systematically studied the effect of anions on the viscosity of IL^30^. Generally, in IL with the cations [Bmim +], the viscosity order with different anions is: ${\upeta }_{[\mathrm{PF}6]-}$> ${\upeta }_{[\mathrm{SbF}6]-}$>${\upeta }_{[\mathrm{BF}4]-}$>${\upeta }_{[\mathrm{TF}2\mathrm{N}]-}$. Although the Bmim[TF2N] has a comparable strong Van der Waals force, its viscosity is the lowest, mainly because its hydrogen bonds are completely suppressed, and thus compensate for the viscosity increasing caused by strong Van der Waals force. In addition, Huddleston et al. analyzed the thermal stability of imidazole IL^31^. It is found that the stability of IL with [TF2N]- anion is higher than that of IL with [BF4]- and [PF6]- anions. Meanwhile, Bansal et al. found that n-butanol can be used as a co-surfactant to reduce the viscosity of microemulsions^32^. Based on previous research, in order to reduce the viscosity and ensure the stability, three kinds of microemulsions (shown in Table 1) have been designed in present work. One type is the system H_2_O/TX-100/Bmim[TF2N] (BT1, BT2 and BT3), while other two types are n-butanol BuOH co-surfactant systems H_2_O/TX-100:BuOH/Bmim[TF2N] (BT1 + BuOH) and H_2_O/TX-100:BuOH/Bmim[PF6] (BF + BuOH). Figure 3 schematically illustrates the structure of the microemulsions. Shown in Fig. 4, the viscosity value of these microemulsions is stable with increasing the shear rate (γ). Interestingly, the newly-designed microemulsions BT1, BT2 and BT3 present a low viscosity of 153mpa·s, 138mpa·s and 114mpa·s, respectively, which are obviously lower than that of microemulsion BF showing a high viscosity of 215mpa·s. After adding the co-surfactant BuOH, the microemulsion systems of BT1 + BuOH and BF + BuOH present an extremely low viscosity of 11mpa·s and 4mpa·s, respectively.

**Figure 5:**
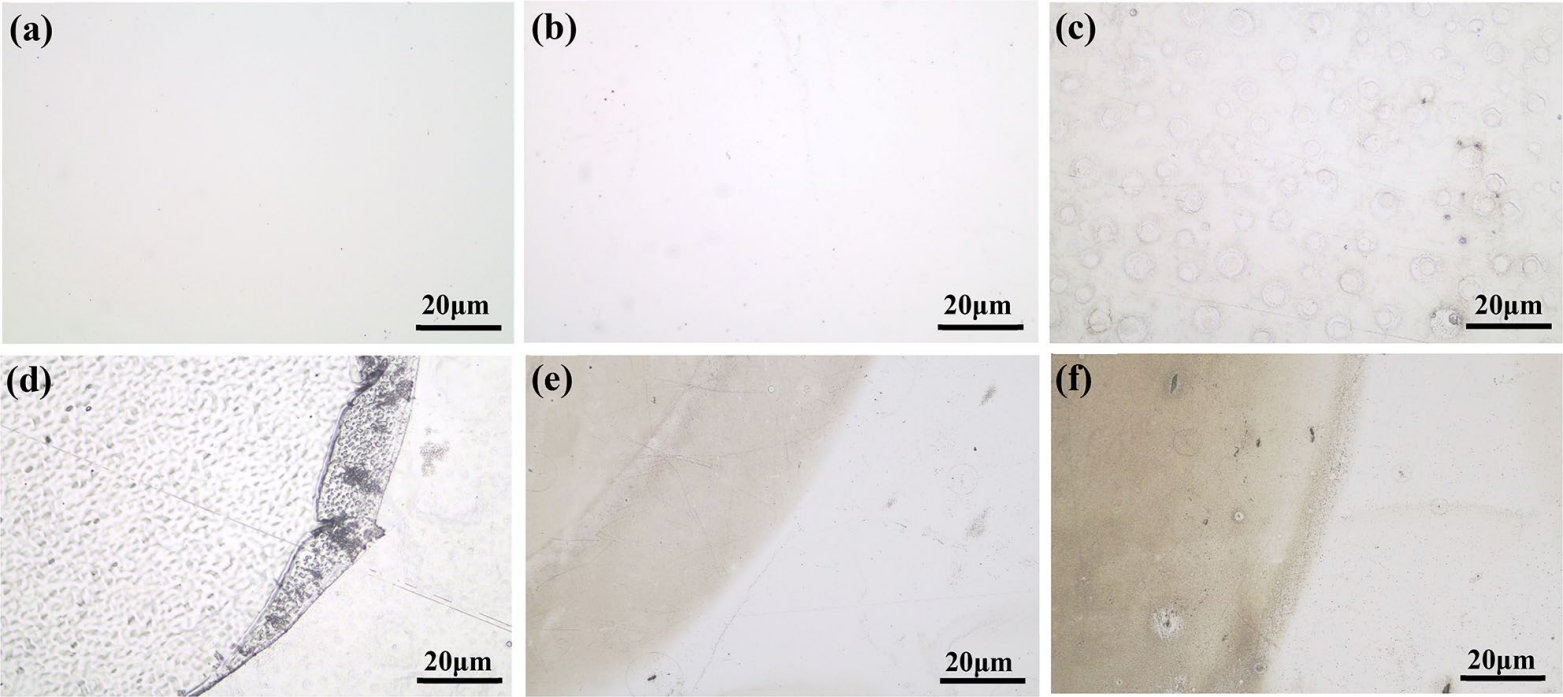
The KDP surface: (**a**) before soaking, (**b**) after soaking in BT1 for 14 h, (**c**) after soaking in BT2 for 14 h, (**d**) after soaking in BT3 for 14 h, (**e**) after soaking in BF + Ba(OH) for 14 h and (**f**) after soaking in BT1 + Ba(OH) for 14 h.

**Figure 6:**
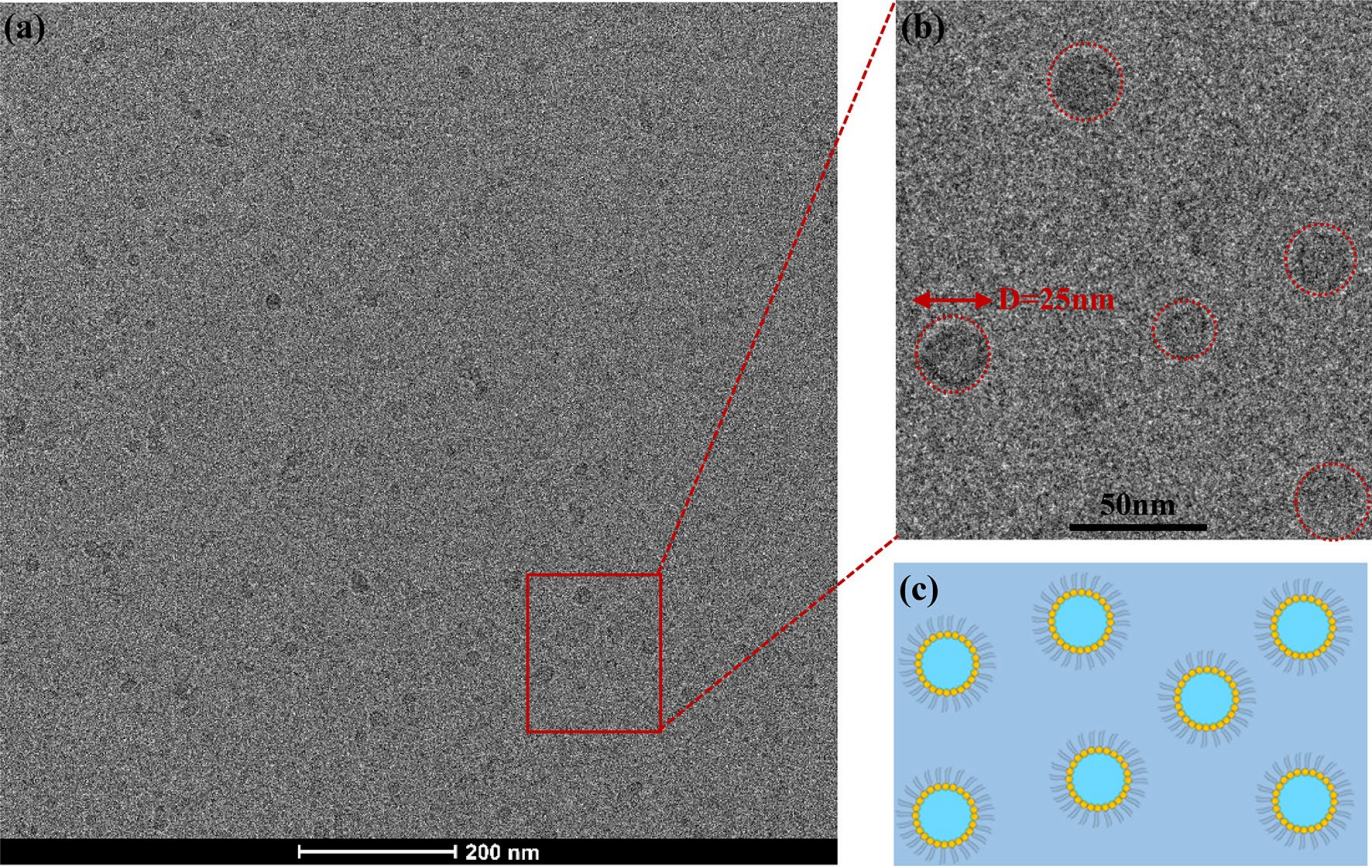
The cryo-TEM images of the microemulsion BT: (**a**) low magnified image, (**b**) high magnified image and (**c**) the schematic illustration of the microemulsion distribution.

The compatibility between these microemulsions and KDP was conducted by placing the microemulsions on the KDP surface. Figure 5a shows the initial KDP surface. Shown in Fig. 5b, as compared to the initial KDP surface, there is almost no deliquescence on the KDP surface in the newly-designed microemulsion BT1. However, shown in Fig. 5c–f, there is an obvious deliquescent phenomenon and yellowish color change on the KDP surface in the microemulsions BT2, BT3, BT1 + BuOH and BF + BuOH. It is supposed that the free water molecules appear in BT2 and BT3, and thus cause serious deliquescence on the KDP surface. In addition, the n-butanol BuOH absorbs large amounts of moisture from the air which also exists in BT1 + BuOH and BF + BuOH in the form of free water molecules, resulting in deliquescence on the KDP surface. Accordingly, the microemulsions BT2, BT3, BT1 + BuOH and BF + BuOH are not suitable for jet polishing, although they present an extremely low viscosity. In present work, basing on above viscosity and compatibility analysis, the newly-designed microemulsion BT1 can be chosen as the potential AFJP fluid. Before the AFJP experiment, it is essential to confirm the structure of the AFJP fluid. In this work, the cryo-transmission electron microscope (cryo-TEM) is used to visually confirm the microemulsion structure. Shown in Fig. 6, according to the TEM results, the AFJP fluid BT1 was concluded to be a typical water-in-oil structure, in which the water cores present an excellent dispersibility and uniformly distribute in the BmimPF6 IL, with a particle size of about 20–25 nm.

**Figure 7:**
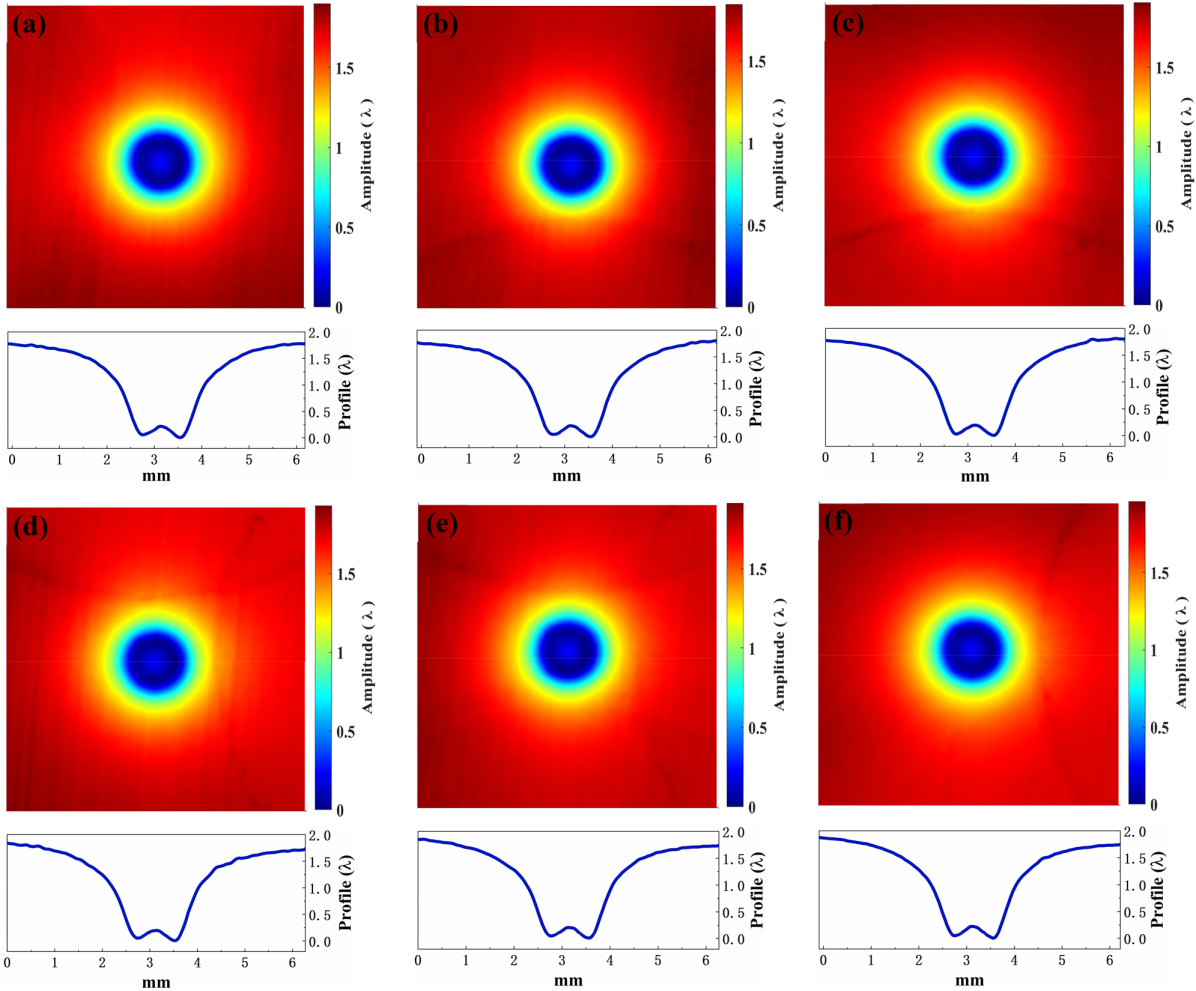
The 3D removal characteristics and corresponded 2D morphology features of jet spots using the BT1 (the time, pressure and nozzle are 5 min, 0.5 MPa and 1 mm, respectively. λ = 632 nm).

**Figure 8:**
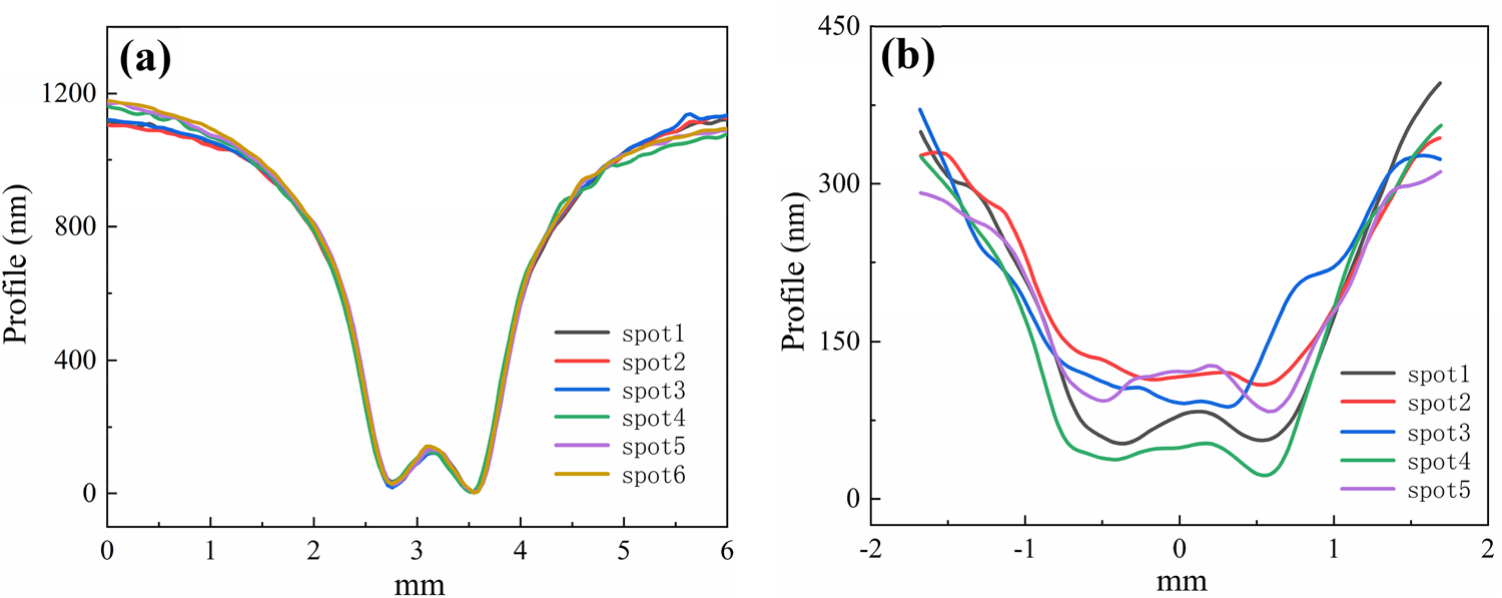
The 2D morphology features of jet spots using (**a**) the BT (the time, pressure and nozzle are 5 min, 0.5 MPa and 1 mm, respectively) and (**b**) the BF (the time, pressure and nozzle are 20 s, 0.5 MPa and 1 mm, respectively^23^).

The controllability and stability of material removal are essential for determining the practicability of the AFJP fluid. For the newly-designed low-viscosity AFJP fluid BT1, a multi-point spot AFJP removal experiment was performed to evaluate the removal controllability and stability. Figure 7 shows the contours and the corresponding cross-sections of these jet spots. It can be clearly seen that the shape of the spot morphology is regular and smooth, presenting a similar Gaussian removal function. Meanwhile, there is no trace of jet fluid flow. Accordingly, it can be concluded that the AFJP fluid BT1 is a controllable and selective non-abrasive jet fluid. Meanwhile, shown in Fig. 8, as compared to the jet spots of previous results which present a high discrepancy when using the AFJP fluid BF^23^, the jet spots when using the low-viscosity AFJP fluid BT1 are nearly the same, indicating a high material removal stability.

Shown in Fig. 8a, the KDP removal rates (maximum value) of these six jet spots using the AFJP fluid BT1 are obtained as 240.1 nm/min, 233.6 nm/min, 241.1 nm/min, 243.8 nm/min, 241.8 nm/min and 247.4 nm/min, respectively. In present work, the dispersion coefficient ${V}_{S}$ is used to evaluate the removal stability which is expressed as follow:

1 $${V}_{S}=S/\overline{x }$$

where $S$ and $\overline{x }$ are the variance and average value of removal rates, respectively, and expressed as follows:

2 $$S=\sqrt{\sum_{i=1}^{n}{\left({x}_{i}-\overline{x }\right)}^{2}/(n-1)}$$

3 $$\overline{x }={\sum }_{i=1}^{n}{x}_{i}/n$$

Based on the removal rates and Eqs. (1), (2) and (3), the dispersion coefficient of removal stability using the low-viscosity AFJP fluid BT1 is calculated to be only 1.9%. However, based on the volumetric removal rates provided in the previous research^23^, the dispersion coefficient using AFJP fluid BF is calculated to be as high as 87.9%. Thereby, it can be concluded that the removal stability is greatly improved by using the low-viscosity AFJP fluid BT1, which is essential for the practical application of AFJP in KDP crystal polishing.

**Figure 9:**
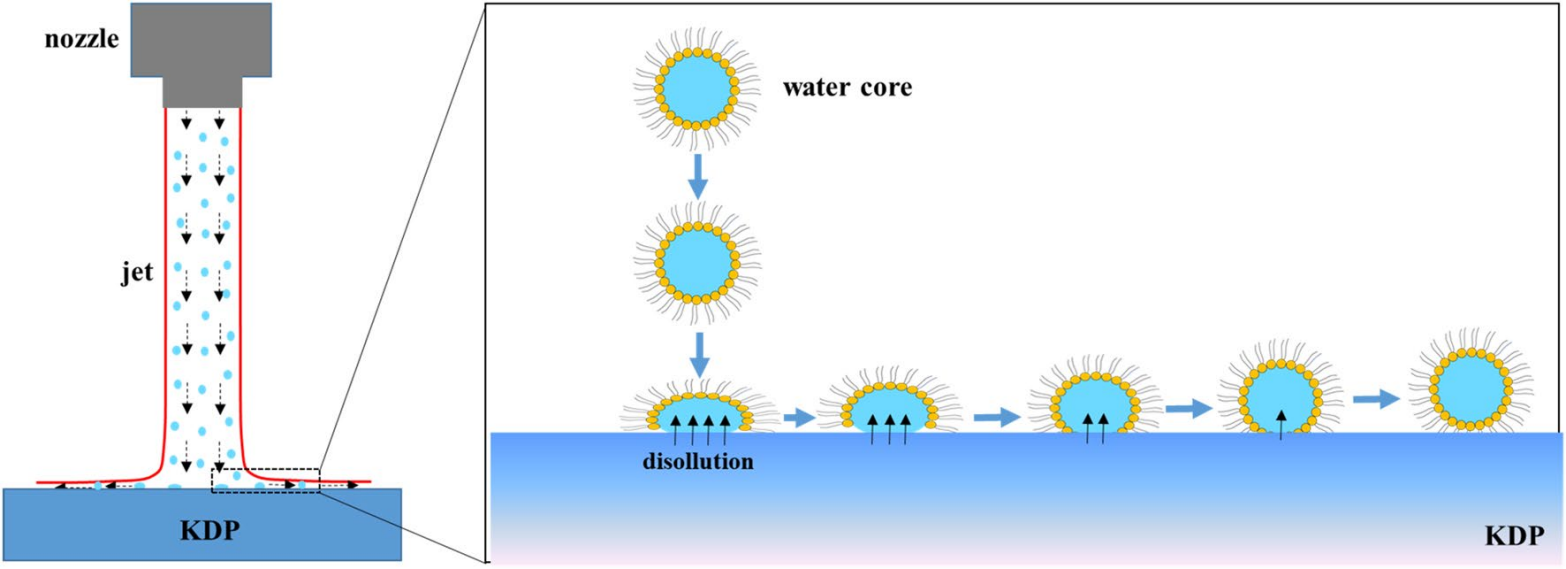
The schematic illustration of removal mechanism of AFJP.

Figure 9 schematically illustrates the removal mechanism of AFJP. So far, it is impossible to directly observe the reforming process of water cores based on the existing methods. Previously, Chen et al. investigated the deforming and reforming process of water cores at the nanoscale using molecular dynamics simulations^33,34^. Based on the simulation and calculation results, it is found that the impact process of nanoscale water cores can be divided into two stages: the deforming stage and reforming stage, when the impact velocity is lower than 665 m/s. At the deforming stage, the droplet undergoes a strong deformation, the spreading radius increases, and the height of the droplet decreases. The initial kinetic and potential energies of the droplet partly transform into the surface free energy. At the end of this spreading stage, the droplet reaches its maximum spreading radius. Afterwards, under the effect of surface tension, the droplet undergoes the reforming stage, the spreading radius of the droplet decreases, and the droplet reverts to the spherical shape. In addition, the same as traditional AJP^35^, due to the spraying and moving of jet fluid, there is a stress field $P\left(x\right)$ and velocity field $v\left(x\right)$ distributed on the KDP surface. After the impact process, the stress caused by the stress field $P\left(x\right)$ keeps the water cores contacting the KDP surface, while the velocity field $v\left(x\right)$ keeps the water cores moving on the KDP surface. Finally, the KDP material is removed by dissolution during the contacting and moving process of the water cores. In dissolution, due to the effect of water molecules, the K^+^, H^+^ and PO4^+^ ions on the KDP surface overcome the interaction force and then diffuse into the water. Accordingly, the removal rate is a function of stress field $P\left(x\right)$ and velocity field $v\left(x\right)$. In previous research, due to the extremely high viscosity of BF, the air is easy to mix into the AFJP fluid, then produces large amounts of bubbles, which significantly affects the stability of the stress field $P\left(x\right)$ and the velocity field $v\left(x\right)$, and thus causes a high instability of the removal function, showing an extremely high dispersion coefficient of 87.9%. In present work, the viscosity of our newly-designed AFJP fluid BT1 is very low, therefore, there is nearly no bubble during a long polishing process, and thus the removal function presents a high stability, with a dispersion coefficient of only 1.9%.

**Figure 10:**
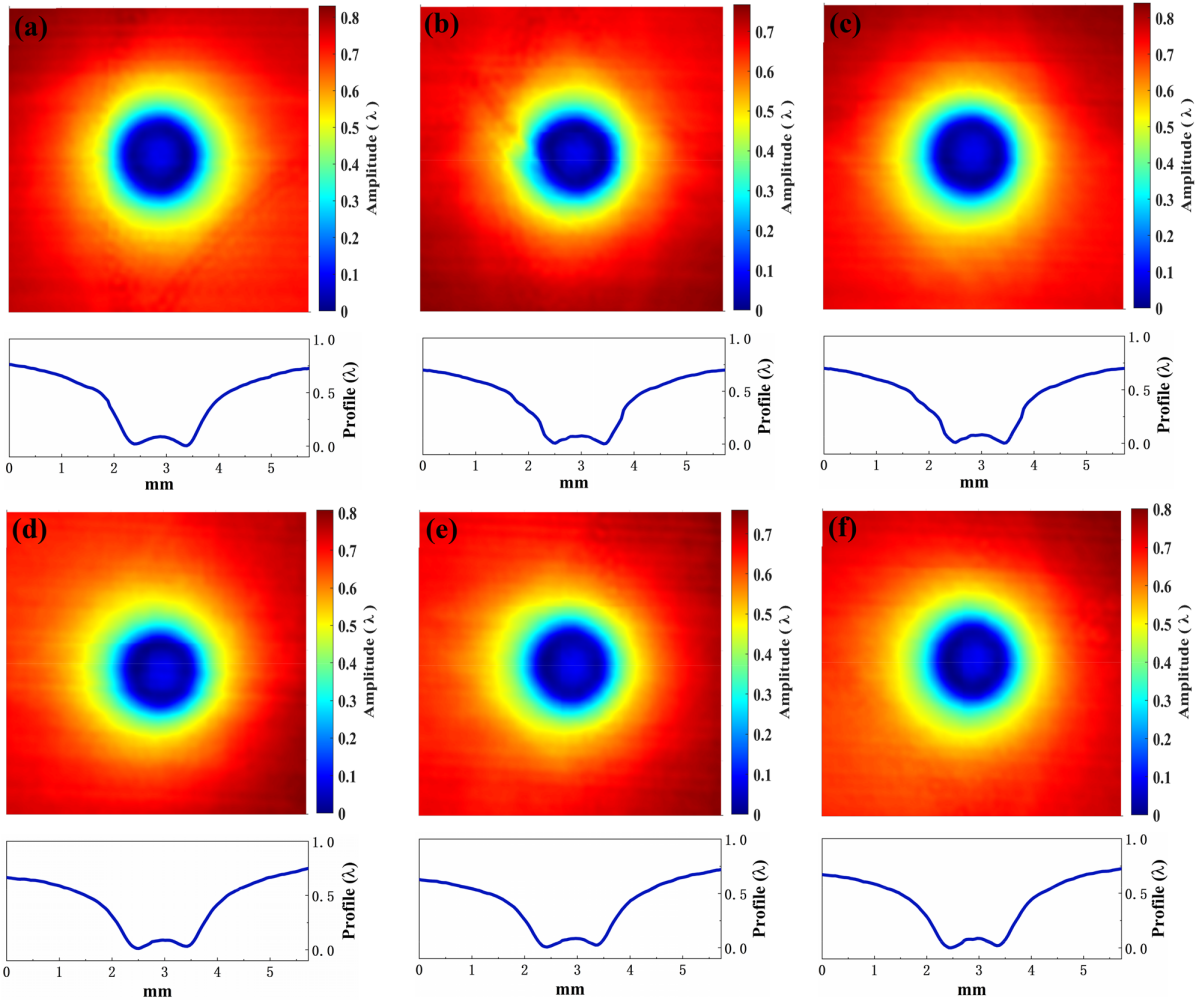
The 3D removal characteristics and corresponded 2D morphology features of jet spots using the BT2 (the time, pressure and nozzle are 1 min, 0.5 MPa and 1 mm, respectively. λ = 632 nm).

**Figure 11:**
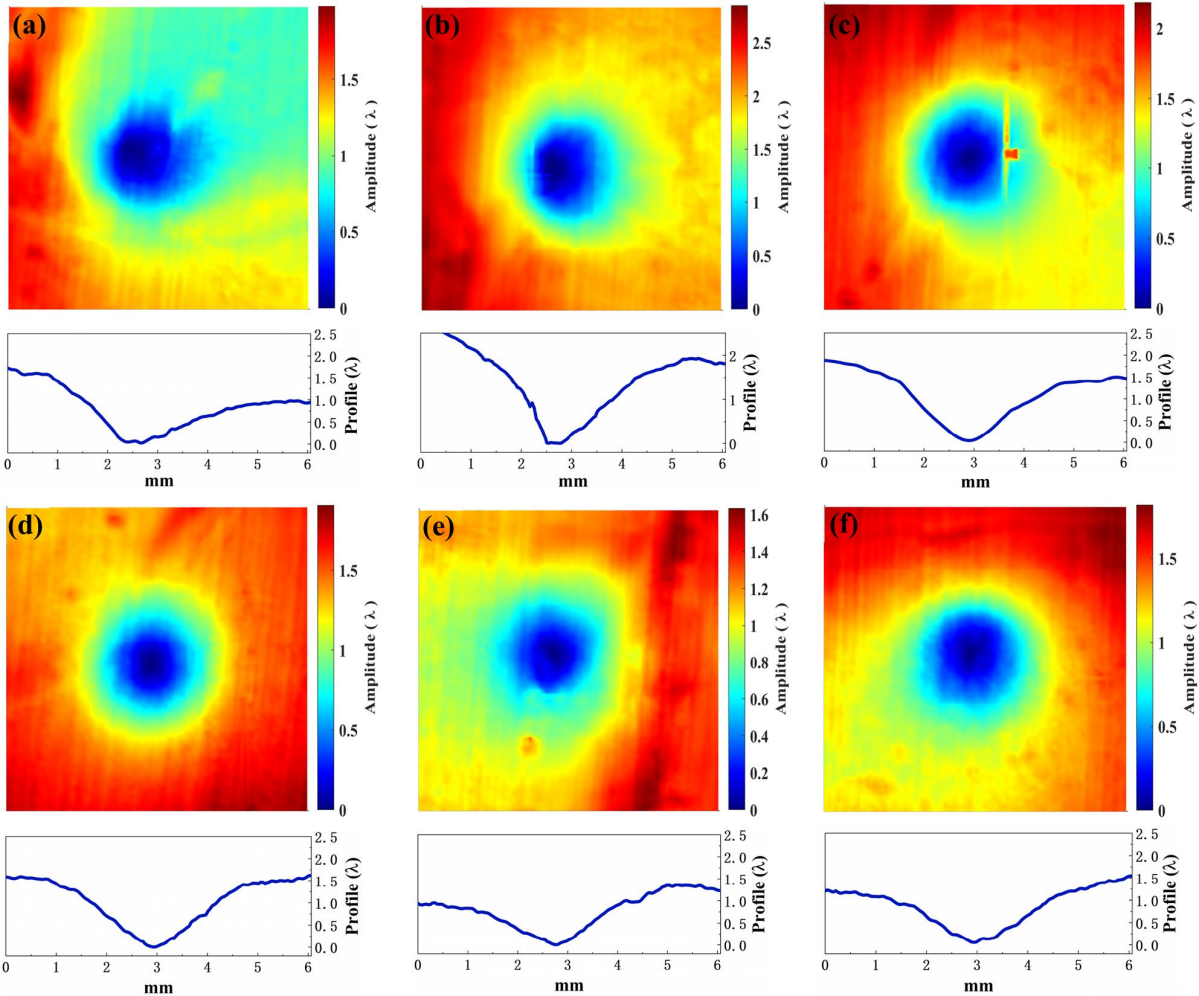
The 3D removal characteristics and corresponded 2D morphology features of jet spots using the BT3 (the time, pressure and nozzle are 10 s, 0.5 MPa and 1 mm, respectively. λ = 632 nm).

**Figure 12:**
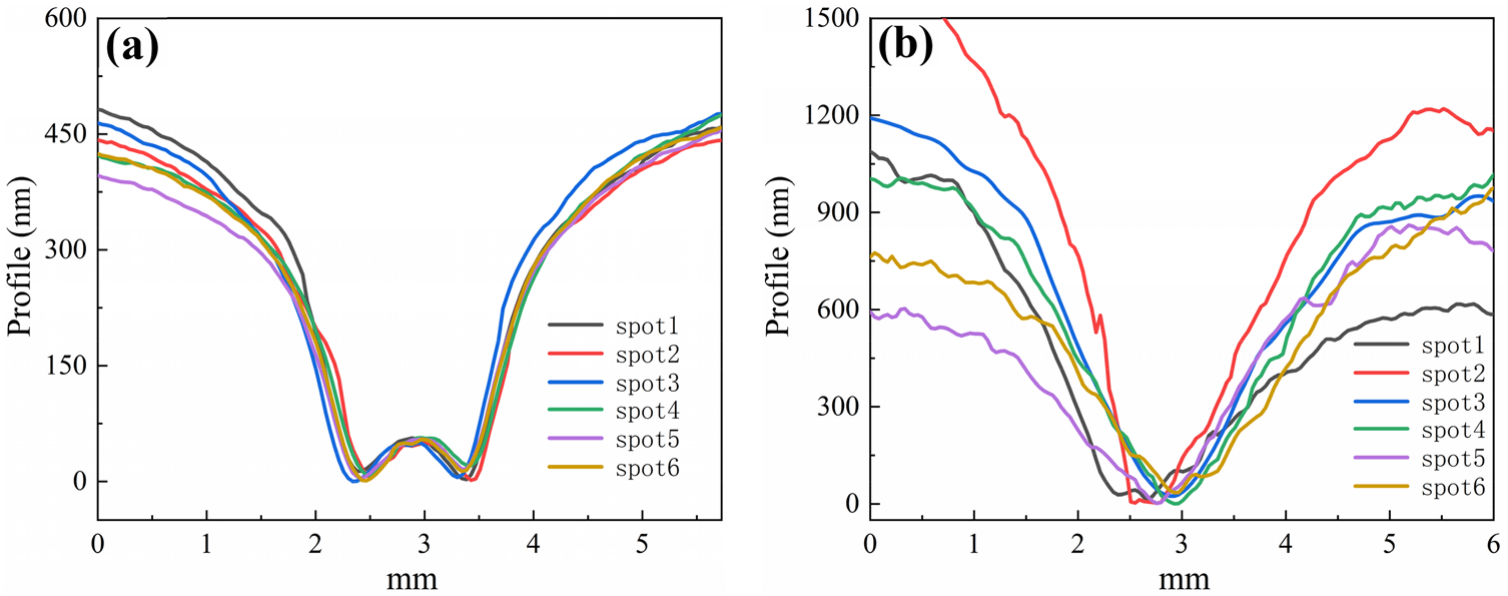
The 2D morphology features of jet spots using (**a**) the BT2 (the time, pressure and nozzle are 1 min, 0.5 MPa and 1 mm, respectively. λ = 632 nm) and (**b**) the BT3 (the time, pressure and nozzle are 10 s, 0.5 MPa and 1 mm, respectively. λ = 632 nm).

In addition, the BT concentration shows a strong influence on the polishing properties. Figures 10 and 11 show the contours and the corresponding cross-sections of jet spots using BT2 and BT3, respectively. As compared to the jet spots using BT1, the removal rate obviously increases when using BT2 and BT3. It is supposed that the increase of water content both increases the diameter and the quantity of water cores and thus increases the removal rate. However, as shown in Fig. 12, the stability of removal function obviously decreases. What’s more, the removal function is no longer presenting a similar Gaussian function when using BT3. It is supposed that the free water molecules in BT2 and BT3 decrease the controllability and stability, especially for BT3, which is no longer a microemulsion that the water and oil separate from each other, resulting in a suspension (shown in the supplementary material).

### Polishing of bulk single-crystal KDP by AFJP

**Figure 13:**
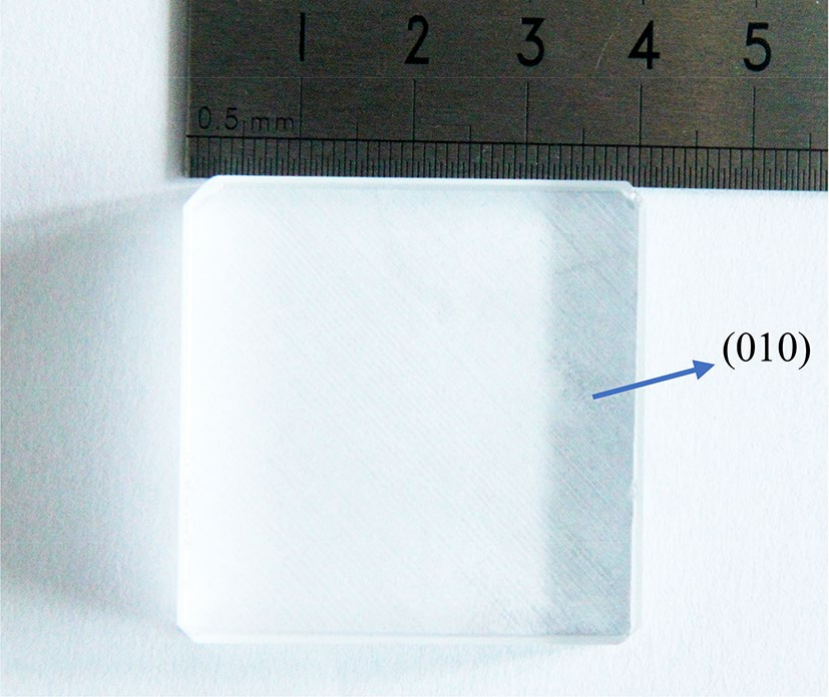
The bulk single-crystal KDP with a dimension of 40 mm × 40 mm × 10 mm.

**Figure 14:**
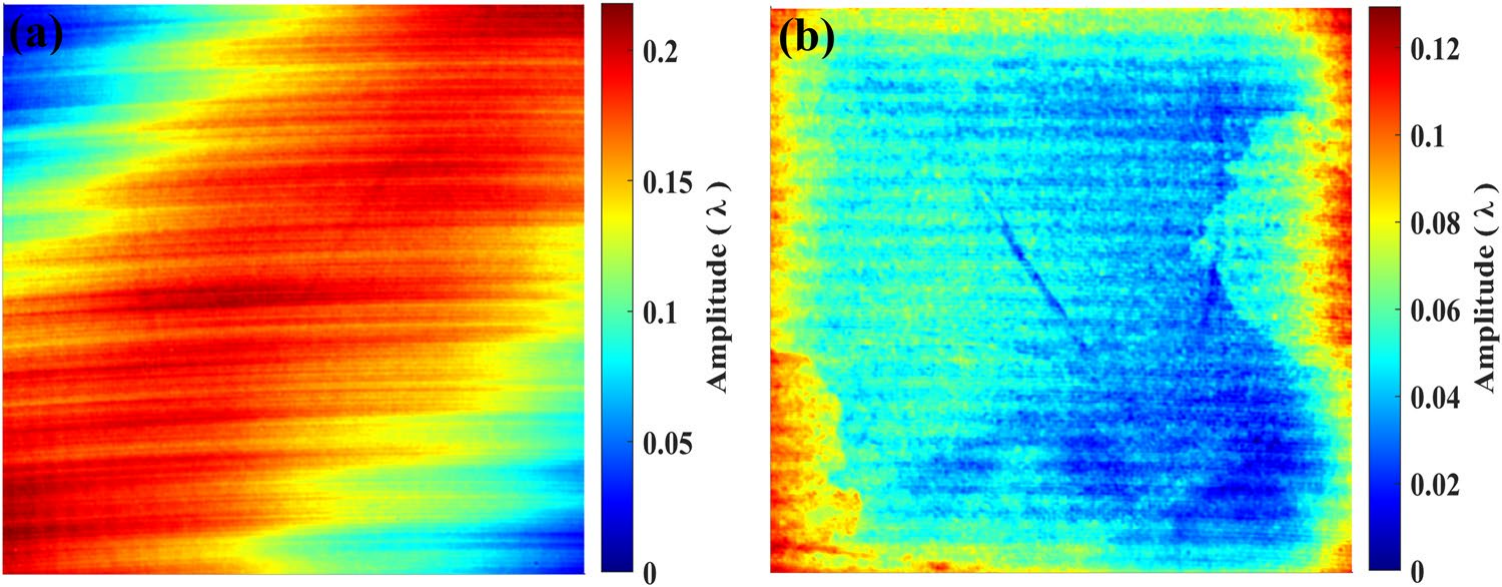
The (010) SPDT surface of the bulk single-crystal KDP with a dimension of 40 mm × 40 mm × 10 mm: (**a**) before AFJP and (**b**) after AFJP using the BT.

In previous research, although the AFJP method is sound novel for mitigating the KDP subsurface damage, the method is limited for bulk single-crystal KDP, due to the extremely high viscosity of AFJP fluid BF^23,24^. During the polishing process, the air is easy to mix into the AFJP fluid BF, then produces large amounts of bubbles, which significantly affects the stability of the stress and velocity field. Thereby, the polishing process has to be stopped after a short polishing time. In present work, we conducted a polishing experiment on the (010) surface of the bulk single-crystal KDP using the low-viscosity AFJP fluid BT1. As shown in Fig. 13, the dimension of the bulk single-crystal KDP is 40 mm × 40 mm × 10 mm. Before polishing experiments, the (010) surface was precisely machined using SPDT. Figure 14a shows the (010) SPDT surface, in which the PV, RMS and Ra are tested to be 137.7 nm, 25.4 nm and 95.7 nm, respectively. After machining, the (010) SPDT surface was polished by AFJP using the low-viscosity BT1 fluid. Figure 14b shows the (010) SPDT surface after AFJP, in which the PV, RMS and Ra are reduced to be 80.5 nm, 11.3 nm and 28.3 nm, respectively. Accordingly, it can be concluded that the surface quality is obviously improved by AFJP.

**Figure 15:**
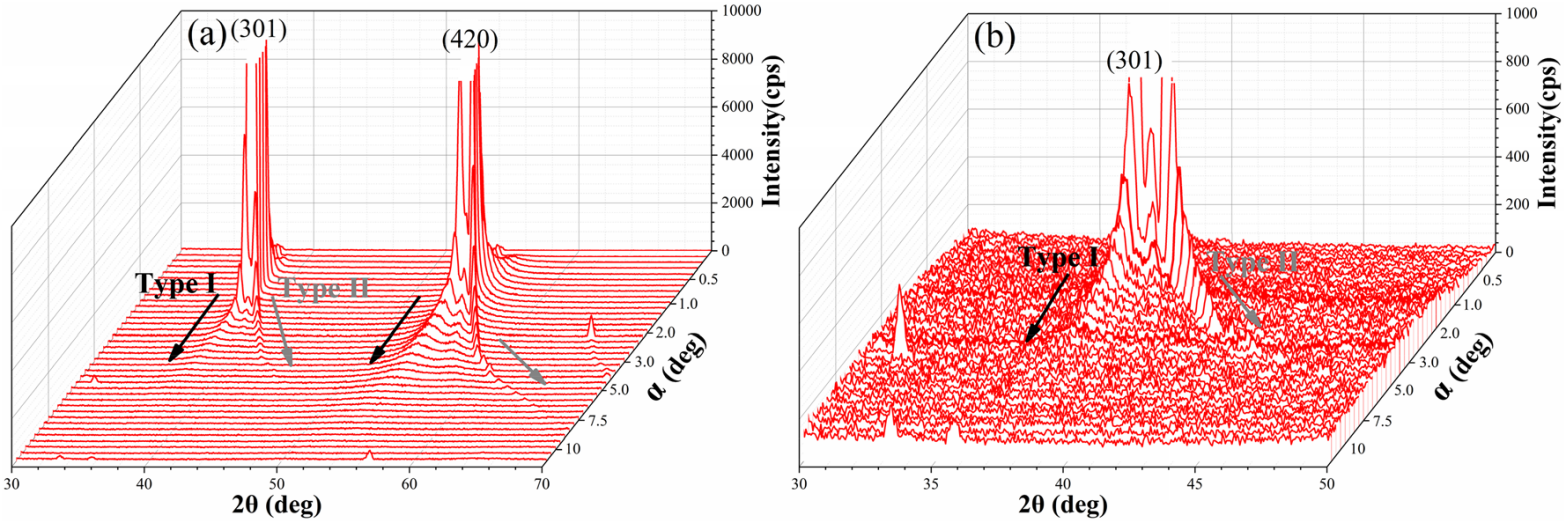
The GIXRD patterns of (010) SPDT surface of bulk single-crystal KDP with increasing incident angle from 0° to 17°: (**a**) the low magnified image and (**b**) the high magnified image.

**Figure 16:**
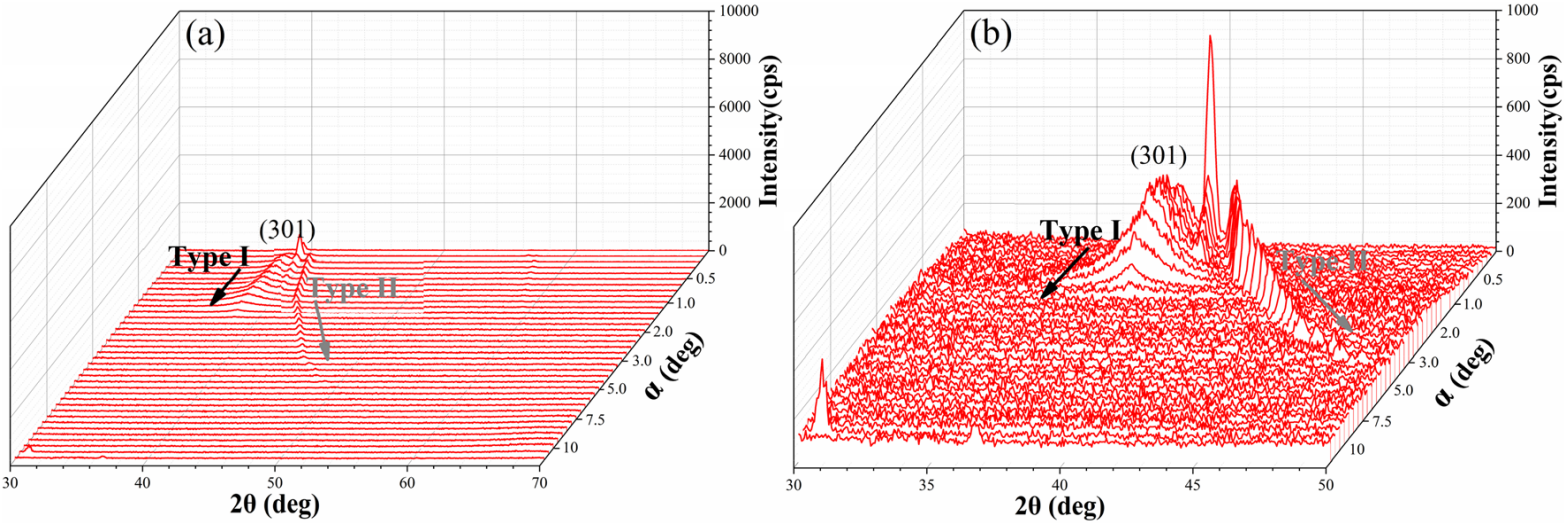
The GIXRD patterns of (010) SPDT surface of bulk single-crystal KDP after AFJP with increasing incident angle from 0° to 17°: (**a**) the low magnified image and (**b**) the high magnified image.

The subsurface damage of bulk single-crystal KDP shows a fatal impact on the optical properties and the laser-induced damage threshold (LIDT) of high-energy laser systems^36,37^. In present work, the mitigation of subsurface damage was evaluated by the GIXRD technique^38–41^. Figure 15 shows the GIXRD patterns of (010) SPDT surface of bulk single-crystal KDP with increasing incident angles from 0° to 17°. The GIXRD patterns reflect the structure information of the subsurface layer, the depth of which increases with increasing incident angle α^42^. As shown in Fig. 15, these diffraction peaks can be divided into two types: the type I is independent of incident angle, while the type II gradually increases with increasing incident angle. The type I consists of (301) and (420) diffraction peaks, which exist at the incident angle ranges of 0° ~ 5.5° and 0° ~ 8.5°, respectively. Interestingly, as shown in Fig. 16, after AFJP on the (010) SPDT surface, the intensity of (301) diffraction peak sharply decreases, and its corresponding incident angle range also decreases to 0° ~ 1.4°, meanwhile, the (420) diffraction peak totally disappears.

**Figure 17:**
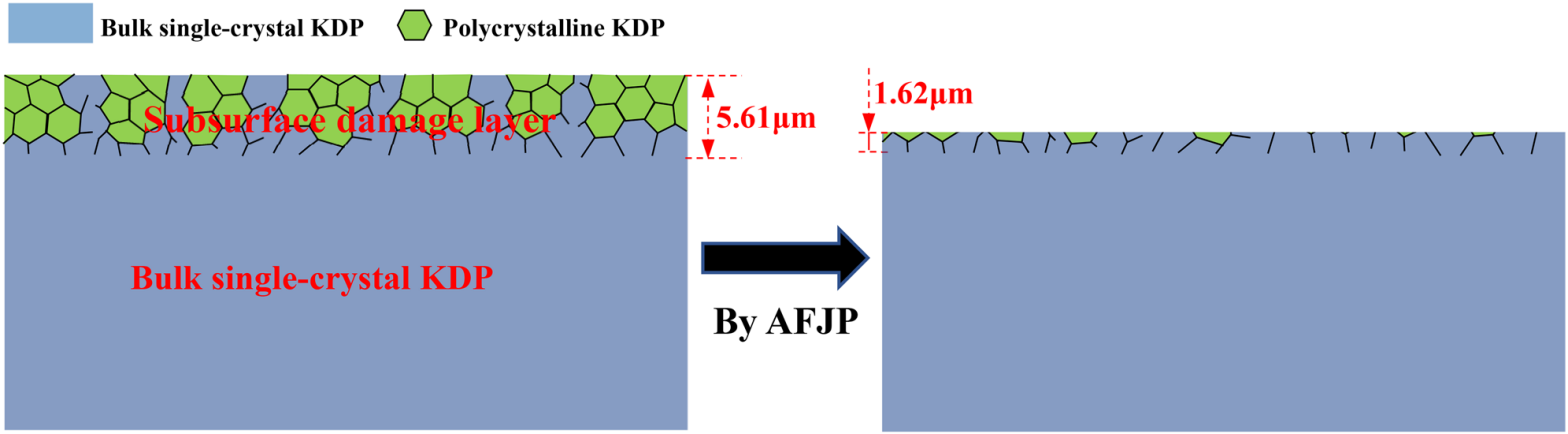
The schematic microstructure of subsurface damage layer of (010) SPDT surface before and after AFJP.

The GIXRD geometry of polycrystalline is different from that of bulk single-crystal. According to the GIXRD geometry, the diffraction peak of polycrystalline is independent of incident angle, while the diffraction peak of bulk single-crystal increases with increasing the incident angle. Therefore, it is supposed that the type I diffraction peaks correspond to the polycrystalline KDP, while the type II diffraction peaks correspond to the bulk single-crystal KDP. Accordingly, it can be concluded that the subsurface damage layer consists of single-crystal matrix and polycrystalline formed by the broken and crack of bulk single-crystal KDP during SPDT, which is schematically shown in Fig. 17. Thereby, the intensity of polycrystalline diffraction peak reflects the damage quantity of subsurface layer. According to the evolution of intensity, it is supposed that the damage quantity of bulk single-crystal KDP is effectively removed by AFJP. In addition, the incident angle range of polycrystalline diffraction peak reflects the thickness of subsurface damage layer. The relationship between the thickness ***t*** of subsurface damage layer and the incident angle $\alpha$ can be expressed as follows^42^:

4 $${\varvec{t}}=\frac{-ln(1-{G}_{t})}{\mu [\frac{1}{sin\alpha }+\frac{1}{\mathrm{sin}(2\theta -\alpha )}]}$$

where $\mu$ is the KDP absorption coefficient of X-ray,$\mu =144.30{cm}^{-1}$ and ${G}_{t}=0.63$. In present work, the incident angle range of (301) diffraction peak is used to evaluate the thickness of subsurface damage layer of (010) SPDT surface before and after AFJP. According to Eq. (4), the thickness of subsurface damage layer is calculated to be 5.61 μm, which obviously decreases to 1.62 μm after AFJP. Therefore, it can be concluded that the subsurface damage layer of bulk single-crystal KDP is effectively mitigated by AFJP.

Accordingly, when using the low-viscosity BT1 fluid, the AFJP method demonstrates its practical application in polishing large-size KDP crystal, which both improves the surface quality and mitigates the subsurface damage.

## Conclusions

In present work, the AFJP of bulk single-crystal KDP was fulfilled at first time, when using a newly-designed low-viscosity microemulsion BT1 as the AFJP fluid. Basing on this research, following can be concluded:

1. The microemulsion BT1 has been designed as the AFJP fluid in present work with a low viscosity of 140mpa·s. The AFJP fluid BT1 is a typical water-in-oil structure, in which the water cores uniformly distribute in the BmimPF6 IL, with a particle size of about 20–25 nm.
2. The low-viscosity AFJP fluid BT1 is a controllable and selective non-abrasive jet fluid, that the shape of the spot morphology is regular and smooth, presenting a similar Gaussian removal function, meanwhile, the dispersion coefficient of the removal rate is only 1.9%.
3. The stress breaks the long-chain surfactant, and keeps the water cores contacting the KDP surface, while the velocity field keeps the water cores moving on the surface, and then the KDP is removed by dissolution during the contacting and moving process of water cores.
4. The AFJP of bulk single-crystal KDP is fulfilled at first time when using the low-viscosity fluid BT1. The surface quality of the bulk single-crystal KDP precisely machined by SPDT, is further improved by AFJP. What’s more, the subsurface damage is obviously mitigated.

## Supplementary Information

Supplementary Information.

## Data Availability

The datasets used and analyzed during the current study are available from the corresponding author on reasonable request.

## References

[1] Li Z, Ge P, Bi W, Liu T, Wang P, Gao Y (2018). Coupling stress caused by thermal and slicing force in KDP crystal slicing with fixed abrasive wire saw. Int. J. Adv. Manuf. Technol..

[2] Demos SG, Raman RN, Yang ST, Negres RA, Schaffers KI, Henesian MA (2011). Measurement of the Raman scattering cross section of the breathing mode in KDP and DKDP crystals. Opt. Express.

[3] Wang D, Li T, Wang S, Wang J, Shen C, Ding J, Li W, Huang P, Lu C (2017). Characteristics of nonlinear optical absorption and refraction for KDP and DKDP crystals. Opt. Mater. Express.

[4] De Yoreo JJ, Burnham AK, Whitman PK (2002). Developing KH_2_PO_4_ and KD_2_PO_4_ crystals for the world’s most powerful laser. Int. Mater. Rev..

[5] Baisden PA, Atherton LJ, Hawley RA, Land TA, Menapace JA, Miller PE, Runkel MJ, Spaeth ML, Stolz CJ, Suratwala TI, Wegner PJ, Wong LL (2016). Large optics for the national ignition facility. Fus. Sci. Technol..

[6] Zhang R, Jia HT, Geng YC, Li P, Liu LQ, Tian XC, Yuan HY, Fan C, Su JQ, Hu DX, Zhu QH, Zheng WG (2016). Research of target uniform illumination on SG-III laser facility. Proc. SPIE.

[7] Chen H, Dai Y, Zheng Z, Gao H, Li X (2011). Effect of crystallographic orientation on cutting forces and surface finish in ductile cutting of KDP crystals. Mach. Sci. Technol..

[8] Liu ZY, Gao H, Guo DM (2019). Polishing technique for KDP crystal based on two-phase air-water fluid. Precis. Eng..

[9] Wang X, Gao H, Yuan JL (2020). Experimental investigation and analytical modelling of the tool influence function of the ultra-precision numerical control polishing method based on the water dissolution principle for KDP crystals. Precis. Eng..

[10] Liu L, Lu L, Gao Q, Zhang R, Chen W (2017). External aerodynamic force on an ultra-precision diamond fly-cutting machine tool for KDP crystal machining. Int. J. Adv. Manuf. Technol..

[11] Wang S, An C, Zhang F, Wang J, Lei X, Zhang J (2016). An experimental and theoretical investigation on the brittle ductile transition and cutting force anisotropy in cutting KDP crystal. Int. J. Mach. Tools Manuf..

[12] Hatefi S, Abou-El-Hossein K (2020). Review of hybrid methods and advanced technologies for in-process metrology in ultra-high-precision single-point diamond turning. Int. J. Adv. Manuf. Technol..

[13] Demos SG, DeMange P, Negres RA, Feit MD (2010). Investigation of the electronic and physical properties of defect structures responsible for laser-induced damage in DKDP crystals. Opt. Express.

[14] Wang S, Wang J, Xu Q, Lei X, Liu Z, Zhang J (2018). Influences of surface defects on the laser-induced damage performances of KDP crystal. Appl. Opt..

[15] Hou N, Zhang Y, Zhang L, Zhang F (2016). Assessing microstructure changes in potassium dihydrogen phosphate crystals induced by mechanical stresses. Scr. Mater..

[16] Menapace JA, Ehrmann PR, Bickel RC (2009). Magnetorheological finishing (MRF) of potassium dihydrogen phosphate (KDP) crystals: Nonaqueous fluids development, optical finish, and laser damage performance at 1064nm and 532nm. Proc. SPIE.

[17] Ji F, Xu M, Wang B, Wang C, Li X, Zhang Y, Zhou M, Huang W, Wei Q, Tang G, He J (2015). Preparation of methoxyl poly (ethylene glycol) (MPEG)-coated carbonyl iron particles (CIPs) and their application in potassium dihydrogen phosphate (KDP) magnetorheological finishing (MRF). Appl. Surf. Sci..

[18] Yang H, Cheng J, Chen M, Wang J, Liu Z, An C, Zheng Y, Hu K, Liu Q (2017). Optimization of morphological parameters for mitigation pits on rear KDP surface: Experiments and numerical modeling. Opt. Express.

[19] Li F, Xie X, Tie G, Hu H, Zhou L (2016). Research on temperature field of KDP crystal under ion beam cleaning. Appl. Opt..

[20] Li F, Xie X, Tie G, Hu H, Zhou L (2017). Figuring process of potassium dihydrogen phosphate crystal using ion beam figuring technology. Appl. Opt..

[21] Wang X, Gao H, Chen YC, Guo DM (2016). A water dissolution method for removing micro-waviness caused by SPDT process on KDP crystals. Int. J. Adv. Manuf. Technol..

[22] Dong H, Wang L, Gao W, Li X, Wang C, Ji F, Pan J, Wang B (2017). KDP aqueous solution-in-oil microemulsion for ultra-precision chemical-mechanical polishing of KDP crystal. Materials (Basel).

[23] Gao W, Wang L, Tian L, Sun P, Dong H, Li X, Wang C, Xu M (2018). Novel abrasive-free jet polishing mechanism for potassium dihydrogen phosphate (KDP) crystal. Opt. Mater. Express.

[24] Gao W, Ji J, Wang C, Wang L, Fan Q, Sun K, Ji F, Xu M (2018). Mitigation of subsurface damage in potassium dihydrogen phosphate (KDP) crystals with a novel abrasive-free jet process. Opt. Mater. Express.

[25] Gao W, Wei QL, Ji JW, Sun PF, Ji F, Wang C, Xu M (2019). Theoretical modeling and analysis of material removal characteristics for KDP crystal in abrasive-free jet processing. Opt. Express..

[26] Gao Y, Han S, Han B, Li G, Shen D, Li Z, Du J, Hou W, Zhang G (2005). TX-100/water/1-butyl-3-methylimidazolium hexafluorophosphate microemulsions. Langmuir.

[27] Yoshimura T, Chiba N, Matsuoka K (2012). Supra-long chain surfactants with double or triple quaternary ammonium headgroups. J. Colloid Interf. Sci..

[28] Khosharay S, Talebi M, Saeed TA, Talaghani SS (2018). Experimental and modeling study of the surface tension and interface of aqueous solutions of alcohols, cetyltrimethylammonium bromide (CTAB) and their mixtures. J. Mol. Liq..

[29] Church J, Willner MR, Renfro BR, Chen Y, Diaz D, Lee WH, Dutcher CS, Lundin JG, Paynter DM (2021). Impact of interfacial tension and critical micelle concentration on bilgewater oil separation. J. Water Process. Eng..

[30] Wasserscheid P, Keim W (2000). Ionic liquids-new solutions for transition metal catalysis. Angew. Chem. Int. Ed..

[31] Huddleston JG, Visser AE, Reichert WM, Willauer HD, Broker GA, Rogers RD (2001). Characterization and comparison of hydrophilic and hydrophobic room temperature ionic liquids incorporating the imidazolium cation. Green Chem..

[32] Bansal VK, Shah DO, Oconnell JP (1980). Influence of alkyl chain length compatibility on microemulsion structure and solubilization. J. Colloid Interface Sci.

[33] Li XH, Zhang XX, Chen M (2015). Estimation of viscous dissipation in nanodroplet impact and spreading. Phys. Fluids.

[34] Li BX, Li XH, Chen M (2017). Spreading and breakup of nanodroplet impinging on surface. Phys. Fluids.

[35] Peng YF, Shen BY, Wang ZZ, Yang P, Yang W, Bi G (2021). Review on polishing technology of small-scale aspheric optics. Int. J. Adv. Manuf. Technol..

[36] Chen M, Li M, Cheng J, Jiang W, Wang J, Xu Q (2011). Study on characteristic parameters influencing laserinduced damage threshold of KH_2_PO_4_ crystal surface machined by single point diamond turning. J. Appl. Phys..

[37] Zhu D, Li Y, Zhang Q, Wang J, Xu Q (2017). Laser-induced damage due to scratches in the surface of nonlinear optical crystals KH_2_PO_4_ (KDP). J. Eur. Opt. Soc.-Rapid.

[38] Marciszko M, Baczmanski A, Braham C, Wrobel M, Wronski S, Cios G (2017). Stress measurements by multi-reflection grazing-incidence X-ray diffraction method (MGIXD) using different radiation wavelengths and different incident angles. Acta Mater..

[39] Jager N, Meindlhumer M, Spor S, Hruby H, Julin J, Stark A, Nahif F, Keckes J, Mitterer C, Daniel R (2020). Microstructural evolution and thermal stability of AlCr(Si)N hard coatings revealed by in-situ high-temperature high-energy grazing incidence transmission X-ray diffraction. Acta Mater..

[40] Tillmann W, Kokalj D, Stangier D, Fu QQ, Kruis FE, Kesper L, Berges U, Westphal C (2021). On the synthesis and structural evolution of artificial CrN/TiN nanocomposites. Appl. Surf. Sci..

[41] Mansilla Y, Arce MD, Gonzalez-Oliver C, Basbus J, Troiani H, Serquis A (2021). Characterization of stabilized ZrO_2_ thin films obtained by sol-gel method. Appl. Surf. Sci..

[42] Wroński S, Wierzbanowski K, Baczmański A, Lodini A, Braham C, Seiler W (2009). X-ray grazing incidence technique-corrections in residual stress measurement-a review. Powder Diffraction Suppl..

