# Supplementary figures and images for: Novel abrasive-free jet polishing for Bulk single-crystal KDP with a low viscosity microemulsion
Source: Sci Rep. 2022 May 18;12:8346. doi: 10.1038/s41598-022-12447-3 (PMC9117678; doi:10.1038/s41598-022-12447-3)

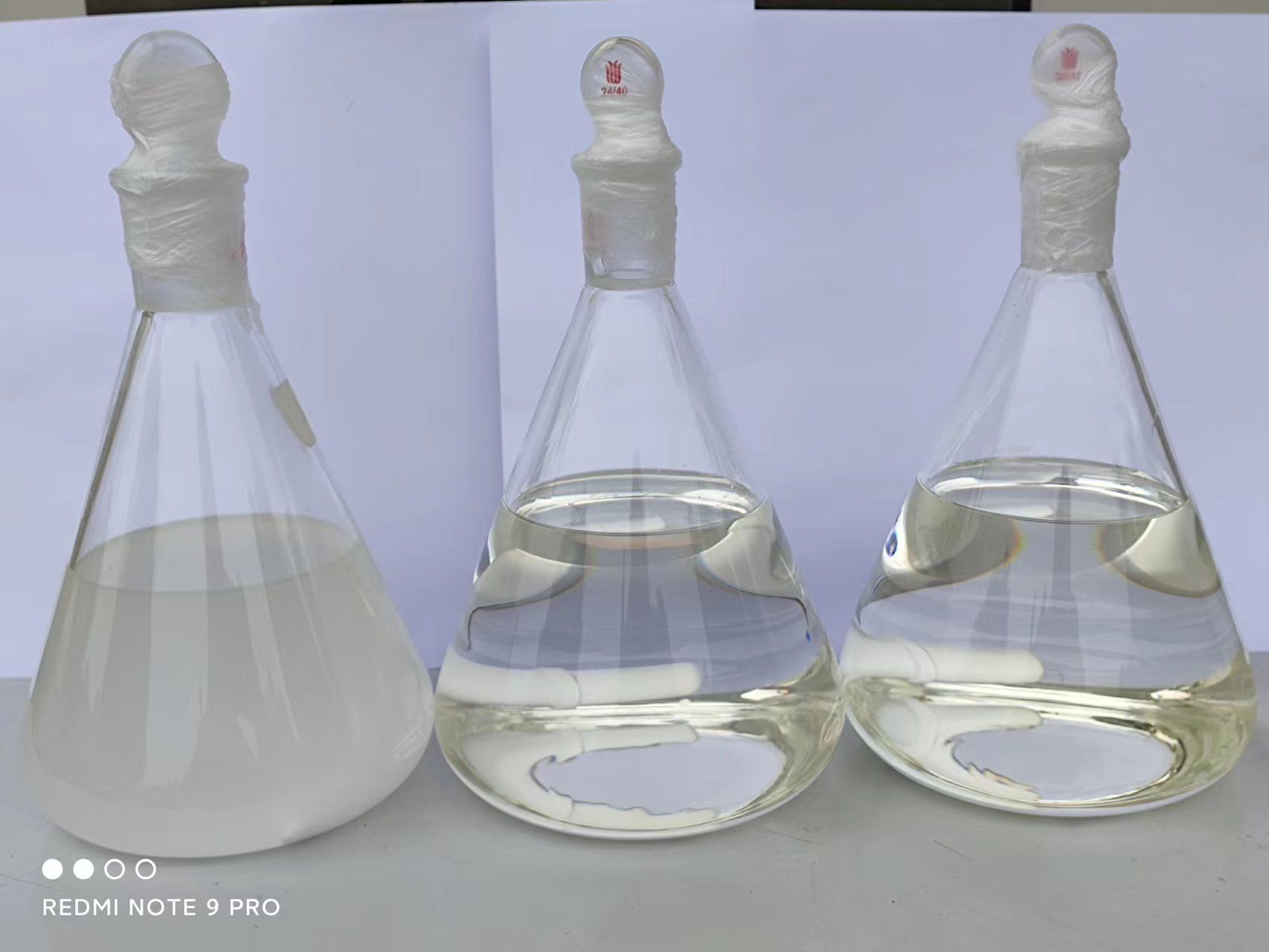


**BT3**

**BT1**

**BT1**

Fig. 1. The BT microemulsions with different water contents: BT1 (2wt.%H2O), BT2 (3wt.% H2O) and BT3 (5wt.% H2O).

Supplement: Supplementary file 1 — Supplementary Information. [file 41598_2022_12447_MOESM1_ESM.docx]
